# Proliferation of Human Primary Myoblasts Is Associated with Altered Energy Metabolism in Dependence on Ageing* In Vivo* and* In Vitro*

**DOI:** 10.1155/2016/8296150

**Published:** 2016-01-05

**Authors:** Reedik Pääsuke, Margus Eimre, Andres Piirsoo, Nadežda Peet, Liidia Laada, Lumme Kadaja, Mart Roosimaa, Mati Pääsuke, Aare Märtson, Enn Seppet, Kalju Paju

**Affiliations:** ^1^Institute of Biomedicine and Translational Medicine, University of Tartu, Ravila 19, 50411 Tartu, Estonia; ^2^Department of Traumatology and Orthopaedics, Tartu University Hospital, L. Puusepa 8, 51014 Tartu, Estonia; ^3^Department of Traumatology and Orthopaedics, University of Tartu, L. Puusepa 8, 51014 Tartu, Estonia; ^4^Institute of Exercise Biology and Physiotherapy, University of Tartu, Ravila 14a, 50411 Tartu, Estonia

## Abstract

*Background*. Ageing is associated with suppressed regenerative potential of muscle precursor cells due to decrease of satellite cells and suppressive intramuscular milieu on their activation, associated with ageing-related low-grade inflammation. The aim of the study was to characterize the function of oxidative phosphorylation (OXPHOS), glycolysis, adenylate kinase (AK), and creatine kinase (CK) mediated systems in young and older individuals.* Materials and Methods*. Myoblasts were cultivated from biopsies taken by transcutaneous conchotomy from vastus lateralis muscle in young (20–29 yrs, *n* = 7) and older (70–79 yrs, *n* = 7) subjects. Energy metabolism was assessed in passages 2 to 6 by oxygraphy and enzyme analysis.* Results*. In myoblasts of young and older subjects the rate of OXPHOS decreased during proliferation from passages 2 to 6. The total activities of CK and AK decreased. Myoblasts of passage 2 cultivated from young muscle showed higher rate of OXPHOS and activities of CK and AK compared to myoblasts from older subjects while hexokinase and pyruvate kinase were not affected by ageing.* Conclusions*. Proliferation of myoblasts* in vitro* is associated with downregulation of OXPHOS and energy storage and transfer systems. Ageing* in vivo* exerts an impact on satellite cells which results in altered metabolic profile in favour of the prevalence of glycolytic pathways over mitochondrial OXPHOS of myoblasts.

## 1. Introduction

Ageing is associated with a progressive loss of skeletal muscle mass and strength referred to as sarcopenia [1, 2]. It is characterized by a decline in the total number of muscle fibers with prevalent atrophy of the type II fibers [3–6]. Despite intensive studies the cellular and molecular mechanisms underlying sarcopenia are still under considerable debate.

Sarcopenia can also be envisaged as an age-dependent impairment of processes responsible for maintenance and repair of the muscle in response to contraction-induced injury [7–10]. These functions largely rely on myogenic stem cells, that is, satellite cells located between the sarcolemma and basal lamina. In response to muscle damage or under increased workload these cells activate, migrate into damaged sites, proliferate, and fuse with existing myofibers or with each other to form new myofibers, whereas a small portion of these cells returns into quiescence [11, 12]. One line of evidence suggests that with ageing the capacities of stem cells to produce their reserve progeny diminish [13]. As a result, the satellite cell number decreases thereby promoting loss of muscle mass [14–23]. Alternatively, ageing modulates the intrinsic properties of the satellite cells characterized by disability to execute the differentiation program [24–27]. It has been demonstrated that aged satellite cells express S100B, an antimyogenic protein stimulating proliferation but inhibiting differentiation [28]. Also, aged satellite cells lose their ability to upregulate the factors involved in the early phases of myogenic differentiation (e.g., MyoD and myogenin) [24]. In contrast to counterparts from young subject's muscle, the satellite cells from aged muscle exhibit the gene expression pattern which favours oxidative damage, altered turnover of cytoskeleton, activation of FOXO-dependent atrophy programs, suppressed myotube formation, and impairment of Ca^2+^-mediated signaling [25–27, 29, 30]. Thus, the increasing evidence indicates resetting of the regulatory systems that lead to altered phenotype of satellite cells in aged muscles.

Interestingly, satellite cells from old muscles also display dysregulation of genes controlling mitochondrial and glycolytic enzymes [31]. The functional consequences of these changes are unclear. Up to now, only the effects of* in vitro* ageing on energy metabolism of cultured myoblasts have been addressed [32, 33]. The results show that replicative senescence results in reduced capacities of myoblasts to differentiate, in association with defective glycose and lipid metabolism, decreased cellular mitochondrial and ATP contents, and increased ROS production, these changes accompanied with normal-to-increased ATP synthesis capacities of mitochondria [32, 33]. These data were recorded in conditions of differentiation of myoblasts, though. It is not clear how ageing* in vitro* affects the energy metabolism in the phase of myoblast proliferation. Also, no study has addressed the status of energy metabolism comparatively in myoblasts cultured from muscle biopsies of older and young subjects. Therefore, the aim of the present study was to investigate the energy metabolism in primary myoblasts derived from muscle biopsies taken from vastus lateralis of young and older subjects. Specifically, the function of the respiratory chain complexes and the system of oxidative phosphorylation (OXPHOS) was assessed in permeabilized myoblasts. Also, the expression and function of various enzymes participating in glycolysis and intracellular energy transfer were investigated.

## 2. Materials and Methods

### 2.1. Chemicals and Reagents

K-Lactobionate, EGTA, and taurine were from Fluka, NADH, pyruvate, and saponin were from SERVA, MgCl_2_ and KCl were from ACROS, and leupeptin was from Roche.

Other reagents, adenine nucleotides (ATP, ADP, and AMP), bovine serum albumin, enzymes (hexokinase, glucose-6-phosphate dehydrogenase, and lactate dehydrogenase), DTT, KH_2_PO_4_, HEPES, L-glutamate, DL-malate, rotenone, succinate, atractyloside, antimycin A, TMPD, ascorbate, EDTA, glucose, Triton X-100, tris, PEP, and NADP were from Sigma.

### 2.2. Human Study Subjects

Seven young subjects (mean age 22.9 ± 1.0 yrs) and 7 older subjects (mean age 76.0 ± 1.8 yrs) were enrolled in the study. Young subjects did not have any record of muscular diseases, traumatic lesions, cardiovascular diseases, or diabetes and were physically active performing some recreational activities but did not train for any specific discipline. Their mean body mass index (BMI) was 22.8 ± 1.0 kg/m^2^. Older subjects also did not have any record of muscular diseases, traumatic lesions, cardiovascular diseases, or diabetes and their mean body mass index (BMI) was 27.4 ± 1.8 kg/m^2^. All investigations were approved by the Research Ethics Committee of the University of Tartu in accordance with the principles of the Declaration of Helsinki (WMA 1997). All subjects signed informed consent based on their voluntary decision and agreement to undergo the procedures. The muscle biopsies were obtained from the vastus lateralis muscle by conchotome technique 10 cm cranially from the superior apex of the patella.

### 2.3. Primary Cultures of Human Myoblasts

Primary cultures of myoblasts were made from human skeletal muscle biopsies using explant culture technique. After 7 to 9 days, cells surrounding the explants were removed with trypsin and propagated in medium (1 vol. DMEM/4 vol. 199 Medium) supplemented with 20% FCS, penicillin (50 units/mL)/streptomycin (50 *μ*g/mL), and 5 ng/mL recombinant human hepatocyte growth factor (HGF) (Pepro Tech). The myoblasts were plated and allowed to proliferate in this medium at 37°C in 5% CO_2_ atmosphere with changing the medium every second day in 100 mm dish. The myoblast purity of human skeletal muscle culture was determined by immunostaining for desmin expression and cultures with purity more than 70% desmin positive cells were used for experiments. After reaching 70–80% confluency the cell populations were trypsinized to establish the next passage. The passages of 2 to 6 were mainly used in the studies of bioenergetic parameters. For these assessments, the cells were removed by trypsin, washed with the medium, counted with hemocytometer, and resuspended in the culture medium supplemented with 10% FCS. The mean doubling time was calculated by counting cell numbers at the beginning (*N*_0_) and end (*N*_*t*_) of the observation period (*t*) and calculated applying the equation dt = *t*/log_2_⁡(*N*_*t*_/*N*_0_), where dt is doubling time and log_2_ is logarithm to base 2.

### 2.4. Determination of Activities of the Respiratory Chain Complexes

The activities of the mitochondrial respiratory chain segments in myoblasts were assessed by polarographic method (Oroboros, Austria) as respiration rates (VO_2_) in Mitomed solution containing (in mM) sucrose 110, K-lactobionate 60, CaK_2_EGTA 0.138, K_2_EGTA 0.362, MgCl_2_ 3, dithiothreitol 0.5, taurine 20, KH_2_PO_4_ 3, K-HEPES 20, pH 7.1, and 1 mg/mL fatty acid free bovine serum albumin (BSA), glutamate, or pyruvate 10 and malate 2, pH 7.1, at 25°C. For permeabilization of the cell membrane, 50 *μ*g/mL saponin was added and the cells were incubated during the 15 min. After addition of 1 mM ADP the respiration was titrated with 10 *μ*M rotenone (Rot) to inhibit complex I, 10 mM succinate (Succ) to activate the complex II dependent respiration, 0.1 mM atractyloside (Atr) to assess the function of adenine nucleotide translocase (ANT), and 10 *μ*M antimycin A (Ant) to inhibit complex III and thereby block the electron flow from complex II to cytochrome c. To activate cytochrome c oxidase 0.5 mM TMPD and 2 mM ascorbate were applied. The rotenone sensitive portion of the NADH-linked ADP-dependent respiration (*V*_ADP_ − *V*_Rot_) was considered to represent the activity of complex I and increment of respiration by succinate (*V*_Succ_ − *V*_Rot_) was taken to represent the complex II activity. The antimycin-sensitive respiration in the presence of atractyloside (*V*_atr_ − *V*_ant_) was considered to represent the proton leak. The COX activity (*V*_Cytox_) was measured as the NaN_3_-sensitive portion of the TMPD-dependent VO_2_.

### 2.5. Determination of the Activities of Kinases

Myoblasts were homogenized by sonication (Bandelin Sonopuls HD 2200, probe MS 72) in homogenization solution containing (in mM) EDTA 1, DTT 1, glucose 10, MgCl_2_ 5, HEPES 5 (pH 8 with NaOH), Triton X-100 0.1%, and leupeptin 5 *μ*g/mL. PK activity measurements were performed in a spectrophotometric cuvette in stirring conditions in solution containing (in mM) tris-HCl 20 (pH 8, 25°C), KCl 15, DTT 0.33, NADH 0.24, PEP 5, ADP 2, and 1 IU/mL LDH. After registration of baseline the reaction was started by addition of the homogenate and from the following changes in NADH oxidation rates at 340 nm the PK activity was calculated. For measurements of AK activity the same solution was used except that ADP was absent and the solution contained 6 IU/mL PK, 0.8 mM PEP, and 3 IU/mL of LDH. After registration of basal CaMgATPase activity in the presence of 1 mM MgATP, 1.3 mM AMP was added to determine the AK activity. For CK activity measurements the homogenates were incubated in a spectrophotometric cuvette in stirring conditions in solution containing (in mM) glucose 20, AMP 20, DTT 0.3, magnesium acetate 3, MgADP 1, NADP 1, tris-HCl 50 (pH 7.4, 25°C), supplemented with 2 IU/mL hexokinase (HK), and 2 IU/mL glucose-6-phosphate dehydrogenase (G6PDH) at 25°C. After stabilization of the optical density, the reaction was started by addition of 20 mM PCr and the rate of NADPH formation was registered. The HK activity was measured in the presence of 2 mM MgATP, 0.6 mM NADP, and 2 IU/mL G6PDH, and the rate of NADPH generation was monitored spectrophotometrically (Perkin-Elmer Lambda 900) after addition of homogenate at 340 nm, 25°C.

### 2.6. Gene Expression Study

In order to study the expression of different isoforms of kinases (CK, AK, and HK), the real-time PCR method was applied. At first, total RNA from 1 × 10^6^ cultured cells (frozen and stored at −80°C) originated from vastus lateralis of older and young subjects was isolated and purified by the RNeasy Plus Mini Kit (Qiagen, Germany) using QIAshredders (Qiagen, Germany) for cell lysate homogenization. To convert RNA (1 *μ*g per reaction) to cDNA, the QuantiTect Reverse Transcription Kit (Qiagen, Germany) that includes genomic DNA elimination reagent was used. Real-time PCR amplification was carried out by using gene-specific primers (Proligo, France, and Oligomer OY, Finland) in QuantiTect SYBR Green PCR Kit (Qiagen, Germany). The process of collecting fluorescence data during PCR was performed by StepOnePlus Real-Time PCR Instrument (Applied Biosystems, USA) using intercalator-based method, also well-known as SYBR Green method. An identical PCR cycle profile was used for all genes. The amplification started by heat activation of HotStarTaq DNA polymerase at 95°C for 15 min. The following 35 cycles of PCR consisted of denaturation step for 15 s at 94°C and primer annealing step for 30 s at 56°C and for 30 s extension phase at 72°C. PCR runs were performed in duplicate or in triplicate and the volume for one reaction was 20 *μ*L.

Determination of relative target quantity in samples was done by the comparative threshold cycle (ΔCT, ΔΔCT) method using StepOne software. Measurements were normalized to multiple endogenous control genes: ACTB, B2M, and HPRT1. The relative quantity of target in each sample was assessed by comparing normalized target quantity in each sample to normalized target quantity in the reference sample. The amplified double-stranded DNA sequences were separated in a 2.4% agarose gel to verify amplicons by length using DNA size marker (100 bp GeneRuler, Fermentas, Lithuania).

### 2.7. Statistical Analysis

Data are given as mean ± SEM. One-way analysis of variance (ANOVA) followed by Scheffe post hoc comparisons was used to test for differences between groups. A level of *p* < 0.05 was selected to indicate statistical significance.

## 3. Results

### 3.1. Characterization of Myoblast Culture Growth

**Figure 1:**
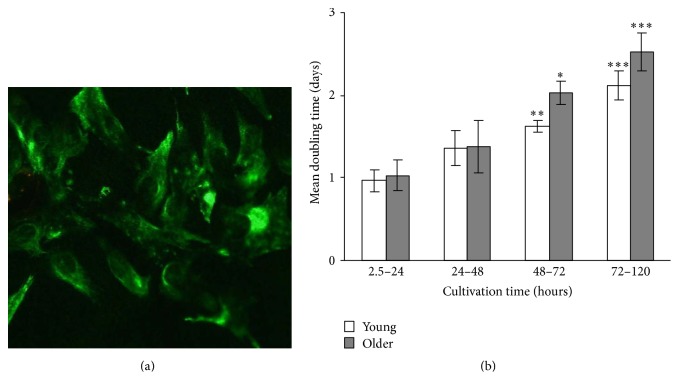
(a) Morphology of desmin-specific (green) skeletal muscle myoblasts of passage 2 in the culture (40x). (b) Proliferation of skeletal muscle myoblasts in culture throughout a single passage for young (*n* = 5) and older (*n* = 7) subjects. The data (mean ± SEM) for cells from passages 3, 5, and 6 were combined, as the dynamics of cell growth was similar within each of the passages. ^*∗*^ *p* < 0.05; ^*∗∗*^ *p* < 0.01; ^*∗∗∗*^ *p* < 0.001 compared to the first day of cultivation.

Figure 1(a) shows a typical morphology of the myoblast culture in proliferation medium in the end of passage 2. Since the medium contained HGF the fibroblasts contamination was maximally suppressed and more than 70% of cells were desmin positive indicating the myogenic nature of the cells. The myogenic purity was similar in the cells obtained from young and older subjects (not shown).

In our conditions the myoblasts reached the 75% confluence during 3–5 days after which they were trypsinized and plated on a new dish. Interestingly, within the given passage the growth rate of the myoblasts was maximum during the first day of cultivation when the number of the cells doubled. Thereafter the growth gradually declined, so that the doubling rate increased twice by the end of incubation. Interestingly, the myoblasts isolated from the older and young subjects grew with similar rate in the beginning of the cultivation. After 3 days the old cells tended to grow slower compared to young ones, but no statistical significance was observed between the groups.

### 3.2. Effect of* In Vitro* and* In Vivo* Ageing on Mitochondrial Function

**Figure 2:**
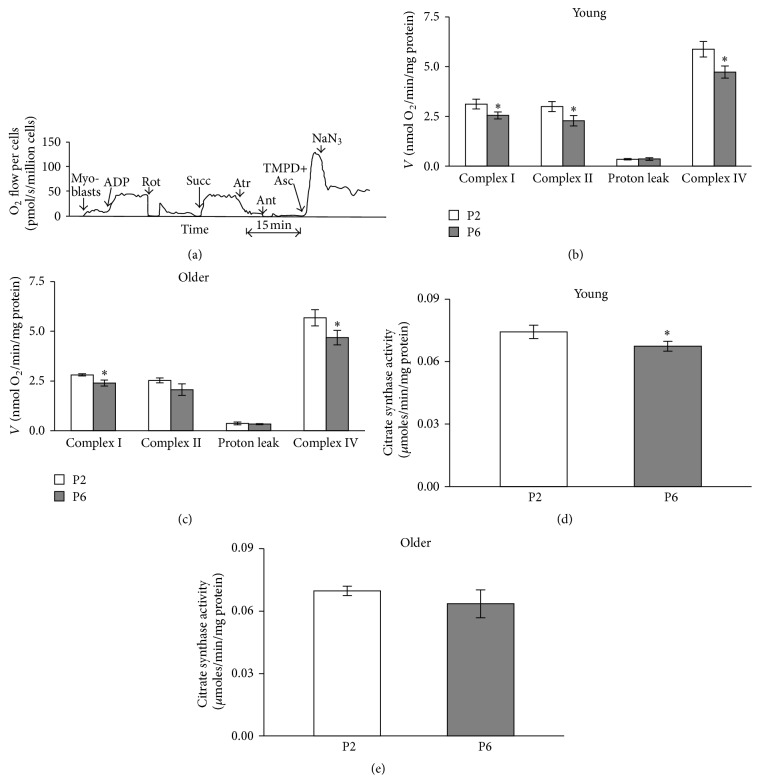
(a) Original recording of the assessment of the respiratory chain function in myoblasts. Myoblasts were incubated in Mitomed medium at 25°C in the presence of 50 *μ*g/mL saponin. Further additions: ADP 1 mM, Rot: 10 *μ*M rotenone, Succ: 10 mM succinate, Atr: 0.1 mM atractyloside, Ant: 10 *μ*M antimycin A, TMPD+Asc: 0.5 mM TMPD with 2 mM ascorbate, and NaN_3_: 5 mM sodium azide. (b, c) Summary of results shown in (a) in groups of cells cultured from young subjects. Complex I: ADP-dependent complex I activity, complex II: ADP-dependent complex II activity, complex IV: complex IV activity, and (d, e) citrate synthase activities in myoblast samples assessed in panel (b). Data are mean ± SEM. ^*∗*^ *p* < 0.05 differences compared to passage 2. P2: passage 2; P6: passage 6.

The function of different respiratory chain complexes in saponin skinned myoblasts was assessed by using a substrate/inhibitor titration protocol (Figure 2(a)). It can be seen that mitochondrial respiration in permeabilized myoblasts strongly increased over the basal levels after addition of ADP in the presence of glutamate and malate. This change indicates tight coupling of complex I dependent respiration to phosphorylation of ADP and effective permeabilization of cell membrane, as ADP exerted only insignificant effect on cellular respiration in the absence of saponin (not shown). In separate experiments (not shown) the concentration of ADP was increased by small increments (cumulatively) to assess the apparent affinity of mitochondria inside the cells. We found that the apparent Km for regulation of OXPHOS with ADP was below 10 *μ*M which corresponds to that parameter in isolated mitochondria [34]. Thus, mitochondria in permeabilized myoblasts were characterized by very high apparent sensitivity towards exogenous ADP which indicates lack of intracellular diffusion restrictions for adenine nucleotides, a property registered earlier for mice cardiac myoblasts [35]. After registration of the respiration in the presence of ADP and NADH-linked substrates (Figure 2(a)) rotenone was added to inhibit complex I. The following addition of succinate reestablished the respiration, now being totally complex II dependent. Next, atractyloside was added that almost totally abolished the succinate dependent respiration. Such a strong control of respiration by atractyloside, an inhibitor of ANT, indicates that the mitochondrial inner membrane remained intact despite the sarcolemmal permeabilization by saponin in myoblasts. In a following step the cytochrome c oxidase was maximally activated by reduction of cytochrome c with TMPD and ascorbic acid and the NaN_3_-insensitive portion of stimulation was used to indicate complex IV dependent respiration. Figures 2(b) and 2(c) show that replicative ageing* in vitro* (from passage 2 to passage 6) is associated with significant decline in the rates of complex I and IV dependent respiration (normalized for cell protein content) in myoblasts cultivated either from young or from older subjects. Similar change was observed also for complex II dependent respiration in young myoblasts, but not in myoblasts from older subjects where only a tendency for decrease was observed. Comparison of the myoblasts from young and older subjects revealed a tendency for decreased respiration rate in the latter group for both complex I and complex II and also for complex IV dependent respiration. In passage 2 these respiration rates in myoblasts from young subjects were 3.12 ± 0.25, 2.99 ± 0.25, and 5.87 ± 0.39 *μ*moles/min/mg protein, respectively. In myoblasts from older subjects respiration rates were 2.80 ± 0.06, 2.53 ± 0.12, and 5.67 ± 0.41 *μ*moles/min/mg protein, respectively. In passage 6 the complex I, complex II, and complex IV dependent respiration rates in myoblasts from young subjects were 2.54 ± 0.18, 2.27 ± 0.26, and 4.73 ± 0.30 *μ*moles/min/mg protein, respectively. In myoblasts from older subjects the respiration rates were 2.40 ± 0.15, 2.07 ± 0.29, and 4.68 ± 0.37 *μ*moles/min/mg protein, respectively. Proton leak was unaffected by ageing either* in vitro* or* in vivo*.

Principally, alterations in ADP-dependent respiration demonstrated in Figure 2(b) may be related to changes in cellular mitochondrial contents or inhibition of the processes of OXPHOS. Assessment of citrate synthase (CS) activity which is proportional to content of mitochondria enables evaluating the role of mitochondrial mass. We found that changes in CS activity roughly paralleled the alterations in respiration; in both young and old cells a decrease during transition from passages 2 to 6 was observed, although statistical significance was observed only in young cells. Normalization of respiration for CS activity (Figure 2(c)) abolished differences due to replicative* in vitro* ageing (not shown).

### 3.3. Effects of Ageing on Expression of Enzymes in Myoblasts

It is plausible that chronological ageing or ageing* in vitro* results in altered balance between the oxidative and glycolytic capacities or between the systems of energy transfer from mitochondria to ATPases. With this assumption the expression of several enzymes at gene and activity levels was determined. Qualitative data of expressions normalized to multiple endogenous control genes, ACTB, B2M, and HPRT1, showed that, in the young passage 2 myoblasts, pyruvate kinase type M2 (PKM2) isoform is most abundantly expressed (1722, *n* = 2). Other enzymes, like cytosolic and mitochondrial AK (qualitative data, 164 and 27, *n* = 2, resp.), brain type CK (105, *n* = 2), and HK isoforms (HK1, 8, *n* = 2, HK2, 11, *n* = 2), are much less expressed. We found that the mitochondrial CK isoenzyme practically is not expressed in myoblasts. At gene levels we could not see significant differences in aged* in vitro* or* in vivo* groups compared to young group.

**Figure 3:**
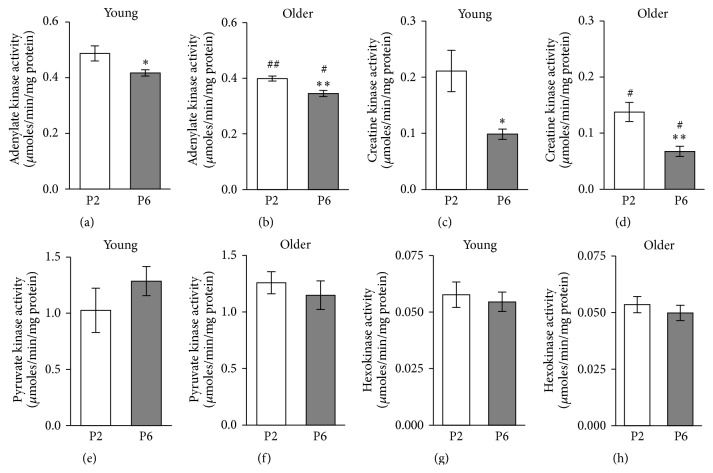
The assessment of the influence of ageing on adenylate kinase (a, b); creatine kinase (c, d); pyruvate kinase (e, f); and hexokinase (g, h) activities in myoblasts of passage 2 (P2) and passage 6 (P6). The myoblasts were derived from the vastus lateralis bioptates taken from young and old subjects. Data are mean ± SEM. ^*∗*^ *p* < 0.05; ^*∗∗*^ *p* < 0.01 compared to passage 2 of myoblasts. ^#^ *p* < 0.05; ^##^ *p* < 0.01 compared to young subjects.

Quantitative assessment of the enzyme activities (Figure 3) revealed that in myoblasts the PK was represented with the highest activity, followed by the activities of AK, CK, and HK, this decreasing order corresponding to the gene expression levels. Nevertheless the activities of PK and AK were ~4 times lower and the activity of HK was ~4 times higher than in muscle cells [36–38].

Based on gene expression data it is conceivable that in myoblasts the high PK activity stems from relatively high expression of PKM2, the AK activity is based mainly on AK1 isoform, and CK activity is based mainly on CKB isoform, whose activity was ~90 times lower than in muscle cells from* m. vastus lateralis* (Linossier et al., 1997). HK activity results from expression of both HK1 and HK2 isoforms.

Interestingly, the activities of AK and CK significantly decreased during replicative senescence of myoblasts from passage 2 to passage 6 in both young and older subjects. Ageing* in vivo* also affected these enzymes as the myoblasts from older subjects exhibited lower AK activity in both passage 2 and passage 6 cells compared to young counterparts (0.399 ± 0.009 and 0.487 ± 0.027 *μ*moles/min/mg protein in passage 2, *p* < 0.05, resp., and 0.345 ± 0.011 and 0.417 ± 0.025 *μ*moles/min/mg protein in passage 6, *p* < 0.05, resp.). Similar* in vivo* ageing effect was observed in CK activity (0.138 ± 0.017 and 0.211 ± 0.027 *μ*moles/min/mg protein, in passage 2, *p* < 0.05, resp., and 0.068 ± 0.009 and 0.098 ± 0.010 *μ*moles/min/mg protein in passage 6, *p* < 0.05, resp.). In contrast, the activities of PK and HK were not influenced by ageing* in vitro* or* in vivo*. However, it is noticeable that the PK activity in myoblasts from young and older subjects responded differently to replicative senescence, with increase and decrease, respectively. As we also have seen a trend to decrease respiratory parameters in* in vivo* aged group of myoblasts we hypothesized that ageing* in vivo* may alter the metabolic profile of the myoblasts. One way to measure this is to assess the ratio of glycolytic to oxidative capacity [39]. Therefore, we have examined the ratios of cellular PK activities as a strongly expressed marker of glycolysis to the maximum OXPHOS activity measured as respiration rate at saturating ADP in conditions of function of different complexes of the respiratory chain (Figure 2).

**Figure 4:**
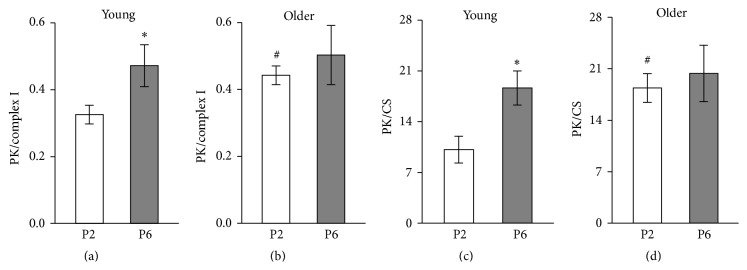
Influence of ageing on activity of pyruvate kinase (PK) normalized for complex I dependent respiration (PK/complex I) in myoblasts of passage 2 (P2) and passage 6 (P6) of young (a) and older (b) subjects. (c-d) Influence of ageing on ratio of activity of PK to citrate synthase (CS) of P2 and P6 of young and older subjects. Data are mean ± SEM. ^*∗*^ *p* < 0.05 compared to passage 2. ^#^ *p* < 0.05 compared to young subjects.

As Figure 4 shows this index increased during* in vitro* ageing in young passage 6 group of cells, but not in the cells from older subjects, because the ratio of PK activity/complex I in the myoblasts from older subjects was already increased (*p* < 0.05) in passage 2 compared to myoblasts from young subjects. Similar differences were observed in the PK/CS ratio (Figures 4(c) and 4(d)).

## 4. Discussion

The present study demonstrated that replicative* in vitro* ageing is associated with significant decline in the rate of ADP-dependent respiration in myoblasts cultivated either from young or from older subjects. The decrease of CS activity was associated with decreased respiration in myoblasts. Minet and Gaster [32] demonstrated that replicative senescence results in decreased mitochondrial mass and ATP content in myotubes while mitochondrial ATP synthesis rate normalized to mitochondrial protein was unchanged. Comparison of the myoblasts from young and older subjects revealed a tendency for decreased respiration rate and CS activity in the latter group. These changes were specifically evident in the earlier passage (2) but disappeared in the later passage (6). Thus, it seems that ageing of subject affects the properties of the satellite cells which determine the function of mitochondria in myoblasts. It is evident that decreased respiratory capacity associated with replicative or* in vivo* senescence of myoblasts resulted from reduced mitochondrial content of the myoblasts.

Gene expression study shows absence of mRNA of the mitochondrial CK isoenzyme, but considerable mRNA level of the mitochondrial isoenzyme AK2 which accounted for 14% of total level of sum of both AK isoforms in myoblasts. These findings suggest that, similarly to the HL-1 cells, myoblasts do not have the energy transport system of CK isoforms; instead AK isoforms participate significantly in energy transport [35]. Our data presents that the activity of AK decreased significantly during replicative senescence of myoblasts, which has not been shown before. Ageing* in vivo* also affected this enzyme as the myoblasts from older subjects exhibited lower AK activity compared to young counterparts. Borges and Essen-Gustavsson [40] and Lanza with coworkers [41] have previously shown that AK activity is decreased also in muscle fibers from older subjects compared to young subjects. As AK also conveys information about the cellular energy state to AMP signal and accordingly metabolic sensors reduce ATP-consuming and activate ATP-generating pathways to adjust energy metabolism and functional activity [42], AK can be an important intermediate step in the mechanism for developing age-related muscle weakness. As AMP signaling can via AMP-activated protein kinase (AMPK) and phosphorylation of PGC-1alpha [43, 44] regulate mitochondrial biogenesis, it is possible that deficit of AK is associated with decrease in the amount of mitochondria of muscle cells in older persons both in ageing* in vivo* and in replicative senescence. Ageing also decreases the differentiation potential of human myoblasts [25]; since the results have already shown that AK-AMPK metabolic signaling axis supports stem cell differentiation [45] our results show significant decrease in AK activity. The decreased AK activity is a significant cause for decreased satellite cell differentiation and hence sarcopenia. Unlike AK-AMPK metabolic signaling, the upstream pathways for expression and activity regulation of AK still remain to be elucidated.

The low CK activities we measured and dominance of BB-CK in myoblasts are consistent with the results of other investigators [46, 47]. In accordance with [48–52] we were unable to show the presence of sarcomeric mitochondrial CK isoenzyme in myoblasts; therefore, we can assume that the CK energy transport system is not developed there. However it is possible that CK is necessary in myoblasts for creating PCr reserve as an energy source for differentiation [51]. Low CK activity still significantly decreased during* in vitro* replicative and* in vivo* senescence, which could also result in decrease in the concentration of PCr and thereby worsening differentiation quality of the muscle fibers.

In Figure 1(b), proliferation of young and older human skeletal muscle myoblasts in culture throughout a single passage showed tendency for longer doubling time, in cultivation times of 48–72 and 72–120 hours, which might be caused by impairment of energy transfer by AK and CK systems as total activities of both enzymes were decreased in* in vivo* aged groups in passage 2 and also in passage 6.

Activity of PK in myoblasts was not influenced by ageing* in vitro* or* in vivo *(Figure 3(e)). But a decline in the activity of PK in human muscles has been reported by several investigators [36, 41]. Also the mitochondrial ATP production capacity and CS activity, indicating number of mitochondria in muscles, become smaller with age [41] but less compared to glycolytic enzymes; hence the muscle becomes more oxidative. It would be interesting to know whether the metabolic profile will change with age, similarly in muscle myogenic cells. We used the ratio of activities of PK and CS as an indicator of metabolic profile. Mean PK activity in dog heart is ~6 times and in vastus lateralis muscle ~26 times higher compared to mean CS activity [52]; human vastuslateralis muscle seems to be somewhat less glycolytic; ratio of mean activities of the abovementioned enzymes is ~17 [53]. Values of PK/CS ratios were similar to PK/CS ratio of human muscle. In young myoblasts from passage 2 PK/CS ratio was 10.1 ± 1.85 and increased significantly during replicative senescence of myoblasts to 18.6 ± 2.35 in passage 6; ageing* in vivo* also affected PK/CS ratio as the myoblasts from older subjects exhibited also a higher PK/CS ratio, 18.4 ± 1.95, in passage 2 compared to young counterparts. For the second measure for evaluation of metabolic profile we used ratio of PK activity/complex I, which, to our knowledge, has not previously been used. The results (Figure 4) show that this ratio in the myoblasts of passage 2 cultivated from older subject's muscle increased compared to myoblasts from young subjects of the same passage. Also the increase in this index was detected during* in vitro* ageing in young passage 6 group of cells, but not in the cells from older subjects. Similarly increased PK activity and glycolysis are shown in cultured human diploid fibroblasts ageing* in vitro* [54]. The most well-known cause for metabolic shift to glycolysis is hypoxia, resulting in binding a nuclear protein, hypoxia-inducible factor 1 (HIF-1), to DNA and increased expression of pyruvate kinase and other glycolytic enzymes [55]. HIF-1 may, however, also cause reprogramming of metabolism to favour glycolysis even under aerobic conditions [56–59]. In H_2_O_2_-induced senescent skin fibroblasts, was observed a decrease in the expression of pyruvate dehydrogenase, activity of cytochrome c oxidase, and the rate of oxygen consumption. The mRNA level of HIF-1 and activities of PDH kinase and lactate dehydrogenase were increased in the senescent skin fibroblasts. These changes in the expression of enzymes suggest a metabolic shift from mitochondrial respiration to glycolysis as a major supply of ATP in these cells [60]. Differently from PK, ratios of HK/complex I and HK/CS in myoblasts were not influenced by ageing* in vitro* nor* in vivo *(data not shown). Likely reason for the lack of change of these ratios is the lower sensitivity of expression of HK to HIF-1.

## 5. Conclusions

The results of the current study demonstrated that proliferation of myoblasts* in vitro* is associated with downregulation of OXPHOS and CK and AK mediated systems of energy storage and transfer. The myoblasts cultivated from older subjects showed lower rate of activities of CK and AK compared to myoblasts from young subjects. The total activities of HK and PK were not affected by ageing* in vivo* or* in vitro*; however there is an uptrend for PK and significantly increased ratio of PK/complex I dependent respiration. Ageing* in vivo* exerts an impact on satellite cells which results in altered metabolic profile in favour of the prevalence of glycolytic pathways over mitochondrial OXPHOS of myoblasts.
